# Comparing two wrist-worn accelerometers (Axivity AX3 and Matrix 003) for measuring movement behaviours in British and Chinese older adults

**DOI:** 10.1123/jmpb.2025-0011

**Published:** 2025-09-26

**Keywords:** raw acceleration, machine learning, physical activity, sleep, data harmonisation, cross-nationality comparison

## Abstract

**Introduction:**

Two nationally representative cohorts, the English Longitudinal Study of Ageing (ELSA) and the China Health and Retirement Longitudinal Study (CHARLS), recently introduced accelerometry to measure movement behaviours. However, the use of different brands (Axivity AX3 and Matrix 003) may hinder data harmonisation. This study assessed whether the raw acceleration data and machine learning-derived physical activity and sleep outcomes were equivalent between these two accelerometers in both British and Chinese adults.

**Methods:**

Eighty-five British and 117 Chinese adults aged ≥50 years wore both accelerometers in a random order on their dominant wrist for up to eight days. Data were processed using UK-derived machine learning algorithms to generate outcomes such as average acceleration (m*g*), time in 24-hour movement behaviours (hours/day), daily step count, and sleep duration (hours/night). Equivalency was assessed using 95% equivalence tests (±10% equivalence zone).

**Results:**

In both British and Chinese adults, average acceleration, sedentary time, time in bed, and sleep duration were equivalent between the two accelerometers, while time in moderate-vigorous physical activity (MVPA) was not (±17.7% in British and ±28.2% in Chinese adults). Time in light physical activity (LPA) was equivalent in British (±6.2%) but borderline in Chinese adults (±10.3%), whereas the opposite was observed for daily step count (±10.5% in British and ±2.9% in Chinese adults).

**Conclusion:**

Average acceleration was comparable between the Axivity AX3 and the Matrix 003 in both British and Chinese adults. Machine learning-derived outcomes were also largely comparable; however, the cross-nationality differences highlight the need for further population-specific algorithm development and validation.

## Introduction

Less time spent being physically active, more time spent being sedentary, and suboptimal sleep duration (outside 6-8 hours per night) are associated with premature mortality and a higher risk of many chronic diseases (Bull et al., 2020; Chaput et al., 2020). However, the majority of evidence to date is based on self-reported measures of these movement behaviours. Although self-report has been sufficient to demonstrate the importance of movement behaviours for health, and can provide valuable contextual information, it lacks the precision to provide specific quantitative recommendations about the optimal amounts and/or composition of movement behaviours for health. For example, studies using accelerometry to examine the association between physical activity and premature mortality have reported effect sizes almost double the size of studies using self-report (Ekelund et al., 2019; Wasfy & Lee, 2022).

An increasing number of large-scale epidemiological cohorts are implementing wrist-worn accelerometry to measure movement behaviours. However, differences in accelerometer brand and processing method create challenges for data harmonisation. The UK Biobank (Doherty et al., 2017), the China Kadoorie Biobank (Chen et al., 2023), and more recently, the English Longitudinal Study of Ageing (ELSA; 2021-23) (Steptoe et al., 2013) all used the Axivity AX3 wrist-worn accelerometer. In contrast, the US National Health and Nutrition Examination Survey (NHANES; 2011-14) used the ActiGraph GT3X+, the British Whitehall II Study (Menai et al., 2017) used the GENEActiv, and the China Health and Retirement Longitudinal Study (CHARLS; 2021-23) (Zhao et al., 2014) developed their own wrist-worn device, the Matrix 003, in collaboration with a Chinese manufacturer. Using open-source software (van Hees et al., 2013; 2014), it is possible to process the raw acceleration data from different accelerometer brands and directly compare them (Rowlands et al., 2019). In addition, there is now the availability of open-source machine learning algorithms to estimate the following: 1) time spent in 24-hour movement behaviours – i.e., moderate-vigorous physical activity (MVPA), light physical activity (LPA), sedentary behaviour (SB), and time in bed (Walmsley et al., 2021); 2) sleep duration and efficiency (Yuan et al., 2024); and 3) step count and cadence (Small et al., 2024). However, it remains to be seen whether these machine learning algorithms can be applied to different accelerometer brands in different populations.

This study aimed to test the first two components of the V3 framework for evaluating Biometric Monitoring Technologies (BioMeTs) such as accelerometers (Goldsack et al., 2020). The primary aim (verification) was to establish whether the raw acceleration data – i.e., the volume and intensity distribution of physical activity – from two different brands of accelerometer (the well-established Axivity AX3 and the newly developed Matrix 003) worn on the dominant wrist can be considered equivalent in both British and Chinese older adults (aged 50 years or older). The secondary aim (analytical validation) was to establish whether the machine learning-derived physical activity and sleep outcomes can also be considered equivalent in both British and Chinese older adults.

## Methods

### Free-living validation study in British adults

A convenience sample of 85 ambulant adults aged 50 years or older living in the UK was recruited by email and word of mouth. All participants provided written informed consent, and the study was approved by the University College London (UCL) Research Ethics Committee. Data were collected between October 2021 and November 2022.

Participants self-reported their height and weight, to calculate body mass index (BMI) in kg/m^2^, and any long-standing (≥3 months) mobility limitations (Supplementary Table S1). Participants were asked to start wearing both accelerometers immediately after receiving them in the post and wear them on their dominant wrist 24 hours per day for eight consecutive days. The positional order of the two accelerometers on the wrist was randomised between participants (Supplementary Figure S1). After eight days, the UCL Courier Service collected the accelerometers. Participants were informed that they could wear the waterproof accelerometers when bathing or swimming but not in extremely high temperature or pressure environments (e.g., in a sauna or when diving). Participants were asked to carry on with their normal activities whilst wearing the accelerometers and did not receive feedback on their activity levels until after they were returned.

### Free-living validation study in Chinese adults

A convenience sample of 117 ambulant adults aged 50 years or older living in China was recruited by visiting two urban and two rural communities in or near Beijing. All participants provided written informed consent, and the study was approved by the Peking University Ethics Review Committee. Data were collected between May and November 2023.

Participants self-reported their height and weight, to calculate BMI in kg/m^2^, and any long-standing (≥3 months) mobility limitations (Supplementary Table S1). The researchers placed both accelerometers on the participants’ dominant wrist in a randomised positional order (Supplementary Figure S1). Participants were asked to wear the accelerometers 24 hours per day for two nights and one day. After the second night, the researchers collected the accelerometers. Participants were informed that they could wear the waterproof accelerometers when bathing or swimming but not in extremely high temperature or pressure environments (e.g., in a sauna or when diving). Participants were asked to carry on with their normal activities whilst wearing the accelerometers and did not receive feedback on their activity levels until after they were collected.

### The Axivity AX3

The Axivity (Axivity Ltd, Newcastle, UK) is a wrist-worn triaxial accelerometer that has been used to measure movement behaviours in large-scale epidemiological cohorts such as the UK Biobank (Doherty et al., 2017), the China Kadoorie Biobank (Chen et al., 2023), and the tenth wave of ELSA (Steptoe et al., 2013). In British adults, the Axivity was set to start recording at 10am two working days after postal dispatch and stop recording seven full days later, whereas in Chinese adults it was set to start recording immediately before placement by one of the researchers and stop recording immediately after collection. In both British and Chinese adults, the Axivity was set to capture triaxial acceleration data at 100 Hz with a dynamic range of ±8 *g*.

### The Matrix 003

The Matrix (Beijing XMatrix Tech. Co., Ltd, Beijing, China) was developed in 2021 to measure movement behaviours in the fifth wave of CHARLS (Zhao et al., 2014). It is a wrist-worn triaxial accelerometer similar to the Axivity, but it also has a gyroscope and a heart rate monitor. It is not possible to configure Matrix accelerometers to start and stop recording at specific times; its only options are to start immediately, after 24 hours, or after 48 hours. Therefore, in British adults, the Matrix was set to start recording just before 10am two working days after postal dispatch, whereas in Chinese adults it was set to start recording immediately before placement by one of the researchers. In both British and Chinese adults, the Matrix was set to capture triaxial acceleration data at 50 Hz with a dynamic range of ±8 *g*.

The Matrix was set to have a lower sampling rate than the Axivity (50 Hz versus 100 Hz) to ensure it had at least seven days of battery life whilst also capturing triaxial gyroscope data at 50 Hz and heart rate data every 15 minutes. However, during data processing, both the Axivity and Matrix datasets were resampled using nearest neighbour interpolation at a rate of 50 Hz. Nearest neighbour interpolation resampling, recommended by Small et al. (2021), was used to avoid unintended smoothing of slower sampled data – specifically, the Matrix data – which has been shown to result in lower overall physical activity.

### Data processing

Each participant’s Matrix dataset was clipped to match their Axivity start and end times. Periods of Axivity non-wear time were then removed from the Matrix time series data and vice versa. Finally, gravity was calibrated in both accelerometers to ensure that at rest, the average magnitude of acceleration was 1 *g* (9.81 m/s^2^).

Participants were excluded if, for at least one of the accelerometers, the data could not be parsed, the device could not be calibrated, more than 1% of readings were ‘clipped’ (fell outside ±8 *g* for *biobankAccelerometerAnalysis*, ±3 *g* for *asleep*, and ±2 *g* for *stepcount*) before or after calibration, or the average acceleration was implausibly high (>100 m*g* for *biobankAccelerometerAnalysis* and *stepcount*, and >200 m*g* for *asleep*).

#### Primary outcomes (verification)

##### Volume and intensity distribution of physical activity

The volume and intensity distribution of physical activity were derived from the Biobank Accelerometer Analysis Tool (github.com/OxWearables/biobankAccelerometerAnalysis, v7.1.1), which was developed and validated by the Oxford Wearables Group (Walmsley et al., 2021). Participants were excluded if they did not have sufficient wear time (≥3 days in British and ≥1 day in Chinese adults, and data in each one-hour period of the 24-hour cycle), with non-wear time defined as unbroken episodes of at least 60 minutes during which the standard deviation (SD) of each axis of acceleration was less than 13 m*g*. To account for potential wear time diurnal bias, recording interruptions and non-wear time were imputed using the average values from the corresponding minute of the day on the remaining days of worn data.

The *biobankAccelerometerAnalysis* algorithm produced the following primary outcomes for each accelerometer separately: total wear time (days), average acceleration (m*g* per day), and time (hours per day) accumulated above incremental acceleration thresholds (>25-200 m*g* in 25-m*g* increments). Average acceleration refers to the Euclidean Norm Minus One (ENMO), a widely used measure of overall physical activity due to its correlation with physical activity energy expenditure (PAEE) (van Hees et al., 2013).

#### Secondary outcomes (analytical validation)

##### Time spent in 24-hour movement behaviours

The *biobankAccelerometerAnalysis* algorithm also produced the following secondary outcomes for each accelerometer separately: time (hours per day) spent in MVPA, in LPA, being sedentary, and in bed.

#### Sleep duration and efficiency

Sleep duration and efficiency were derived from a sleep staging algorithm (github.com/OxWearables/asleep, v0.4.13), which was also developed and validated by the Oxford Wearables Group (Yuan et al., 2024). The *asleep* algorithm classifies each 30-second epoch of acceleration data into one of the three sleep stages: 1) wake; 2) rapid eye movement sleep (REM); and 3) non-rapid eye movement sleep (NREM). Participants were excluded if they did not have sufficient wear time (≥22 hours per day for ≥3 days [including ≥1 weekend day] in British and ≥1 day in Chinese adults), with non-wear time defined as unbroken episodes of at least 90 minutes during which the SD of each axis of acceleration was less than 13 m*g*.

For each accelerometer separately, the *asleep* algorithm calculated the following sleep parameters for the longest sleep window over a noon-to-noon interval, with up to 60 minutes of sleep discontinuity allowed: overnight sleep duration (hours per night) and sleep efficiency (percentage of time in bed spent asleep).

#### Step count and cadence

Step count and cadence were derived from a hybrid machine learning and peak detection step counting algorithm (github.com/OxWearables/stepcount, v3.8.0), which was also developed and validated by the Oxford Wearables Group (Small et al., 2024). Participants were excluded if they did not have sufficient wear time (≥3 days in British and ≥1 day in Chinese adults, and data in each one-hour period of the 24-hour cycle), with non-wear time defined as unbroken episodes of at least 90 minutes during which the SD of each axis of acceleration was less than 13 m*g*. To account for potential wear time diurnal bias, recording interruptions and non-wear time were imputed using the average values from the corresponding minute of the day on the remaining days of worn data.

The *stepcount* algorithm produced the following secondary outcomes for each accelerometer separately: overall daily step count (steps per day) and peak cadence (steps per minute). Overall daily step count was reported as the median number of steps taken per day across the monitoring period. One-minute peak cadence was calculated as previously described by Saint-Maurice et al. (2020).

### Statistical analyses

Descriptive statistics (mean [SD] where data were normally distributed, otherwise median [25^th^-75^th^ percentile]) were calculated for all outcomes, with differences between British and Chinese adults being examined using the independent *t*-test or the Chi-squared test, and differences between the two accelerometer brands being examined using the paired *t*-test or the Wilcoxon signed-rank test.

We used 95% equivalence tests with a 10% equivalence zone to determine whether the 95% confidence interval (95% CI) for the mean of one accelerometer fell within ±10% of the mean of the other accelerometer (Wellek, 2003). This was based on a previous study comparing three different accelerometers (Axivity AX3, ActiGraph GT9X, and GENEActiv) worn on both wrists (Rowlands et al., 2019), as well as many other validity studies of physical behaviour measures (O’Brien, 2021). Where data were not normally distributed, the log transformation of the original data were used for the equivalency analyses. As neither accelerometer can be considered the gold standard, equivalency analyses were carried out twice – i.e., with each accelerometer as the reference monitor. In all cases, equivalency was consistent regardless of the reference monitor. Therefore, results are presented with the Axivity as the reference monitor because it is more established.

The level of agreement between outputs from the two accelerometers was determined using intraclass correlation coefficients (ICCs; two-way mixed effects, absolute agreement, single measures) with 95% CI, and mean bias with 95% limits of agreement (LoA) (Bland & Altman, 1986). The ICC was classified as ‘poor’, ‘moderate’, ‘good’, or ‘excellent’ reliability if the lower band of the 95% CI of the ICC was <0.5, 0.5-0.75, >0.75-0.9, or >0.9, respectively (Koo & Li, 2016).

Separate analyses were conducted for British and Chinese adults, and all analyses were conducted in RStudio (R version 4.4.1). Evidence of suggestive statistical significance was defined as *p* < 0.05.

## Results

Of the 85 participants who were recruited in the UK, 82 had valid data for all outcomes from both accelerometers (52 [63.4%] female, mean [SD] age: 65.6 [10.2] years, 54 [65.9%] living in an urban area, mean [SD] BMI: 24.4 [3.6] kg/m^2^, 21 [25.6%] with mobility limitations). Of the 117 participants who were recruited in China, 106 had valid data for all outcomes from both accelerometers. The Chinese participants had similar characteristics to the British participants, except they were more likely to have mobility limitations (64 [61.0%] female, mean [SD] age: 66.7 [10.3] years, 58 [55.2%] living in an urban area, mean [SD] BMI: 24.4 [3.2] kg/m^2^, 49 [46.7%] with mobility limitations). Descriptive statistics for all outcomes by nationality (British or Chinese) and accelerometer brand (Axivity or Matrix) are presented in Table 1.

In both British and Chinese adults, time spent in bed and sleeping were higher for the Matrix than the Axivity, whilst sleep efficiency and sedentary time were lower. In British adults, overall daily step count was lower for the Matrix than the Axivity, whereas the opposite was observed in Chinese adults. In British adults only, total wear time was higher for the Matrix than the Axivity, whilst overall physical activity (i.e., average acceleration), time accumulated above the lowest threshold of acceleration (25 m*g*), and time spent in LPA were lower. In Chinese adults only, time accumulated above higher thresholds of acceleration (>100 m*g*) were higher for the Matrix than the Axivity.

### Verification results

Equivalency results for the volume and intensity distribution of physical activity by nationality are shown in Figure 1 and Supplementary Table S2. In Figure 1, markers are denoted in solid squares if the 95% CI for the mean of the Matrix 003 fell within ±10% of the mean of the Axivity AX3, otherwise markers are denoted in hollow squares. In both British and Chinese adults, total wear time, average acceleration, and time accumulated above the two lowest thresholds of acceleration (25 and 50 m*g*) were equivalent between the two accelerometer brands (within ±10% equivalence zone). Time accumulated above 75 m*g* was borderline equivalent in British (±10.33%) but not equivalent in Chinese adults (±14.33%), and time accumulated above the five highest thresholds of acceleration (100-200 m*g*) were not equivalent in either British or Chinese adults.

Agreement results for the volume and intensity distribution of physical activity by nationality are shown in Figure 2 and Supplementary Table S3. Bland-Altman plots are also shown in Supplementary Figure S2. In Figure 2, markers are denoted in solid squares if the lower band of the 95% CI of the ICC was good (>0.75) or excellent (>0.9), otherwise markers are denoted in hollow squares. In both British and Chinese adults, reliability between the two accelerometer brands was excellent for total wear time, average acceleration, and time accumulated above all thresholds of acceleration (25-200 m*g*).

### Analytical validation results

#### Time spent in 24-hour movement behaviours

Equivalency results for time spent in 24-hour movement behaviours by nationality are shown in Figure 1 and Supplementary Table S2. In both British and Chinese adults, time spent in bed and being sedentary were equivalent between the two accelerometer brands (within ±10% equivalence zone); whereas time spent in MVPA was not equivalent, particularly in Chinese adults (±17.74% in British and ±28.20% in Chinese adults). Time spent in LPA was equivalent between the two accelerometer brands in British (±6.22%) but only borderline equivalent in Chinese adults (±10.28%).

Agreement results for time spent in 24-hour movement behaviours are shown in Figure 2 and Supplementary Table S3. Bland-Altman plots are also shown in Supplementary Figure S2. In both British and Chinese adults, reliability between the two accelerometer brands was excellent for time spent in both LPA and MVPA but only moderate for time spent in bed. Reliability between the two accelerometer brands was excellent for sedentary time in British but only good in Chinese adults.

#### Sleep duration and efficiency

Equivalency results for sleep duration and efficiency are shown in Figure 1 and Supplementary Table S2. In both British and Chinese adults, both overnight sleep duration and sleep efficiency were equivalent between the two accelerometer brands (within ±10% equivalence zone).

Agreement results for sleep duration and efficiency are shown in Figure 2 and Supplementary Table S3. Bland-Altman plots are also shown in Supplementary Figure S2. In both British and Chinese adults, reliability between the two accelerometer brands was good for sleep efficiency. Reliability between the two accelerometer brands was good for overnight sleep duration in British but poor in Chinese adults.

#### Step count and cadence

Equivalency results for step count and cadence are shown in Figure 1 and Supplementary Table S2. In both British and Chinese adults, peak cadence was equivalent between the two accelerometer brands (within ±10% equivalence zone). Overall daily step count was also equivalent between the two accelerometer brands in Chinese (±2.93%) but only borderline equivalent in British adults (±10.50%).

Agreement results for step count and cadence are shown in Figure 2 and Supplementary Table S3. Bland-Altman plots are also shown in Supplementary Figure S2. In both British and Chinese adults, reliability between the two accelerometer brands was excellent for both overall daily step count and peak cadence.

## Discussion

In both British and Chinese adults, average acceleration was equivalent between the Axivity AX3 and the Matrix 003. The vast majority of machine learning-derived physical activity and sleep outcomes were also equivalent or borderline equivalent. The only exceptions were time spent above higher thresholds of acceleration (>75 m*g*), including time spent in MVPA. However, there were a few notable cross-nationality differences. In Chinese compared to British adults, equivalency between the two accelerometer brands was lower for time spent in both LPA and MVPA but higher for overall daily step count. Furthermore, the ICCs were good or excellent (>0.75) for all outcomes, except for time spent in bed in both British and Chinese adults and overnight sleep duration in Chinese adults only.

We found that average acceleration was equivalent between the Axivity AX3 and the Matrix 003 when measured at the dominant wrist. In contrast, in 56 young British adults (mean age 24.5 years), Rowlands et al. (2019) found that average acceleration measured at the dominant wrist was approximately 10% higher for the Axivity AX3 and the GENEActiv than for the ActiGraph GT9X. Our results suggest that accelerometry data from the UK Biobank, China Kadoorie Biobank, ELSA, and CHARLS can be pooled and/or compared because they all used the Axivity AX3 or the Matrix 003 on the dominant wrist. We also found that time spent above lower thresholds of acceleration (≤75 m*g*) were equivalent between the Axivity AX3 and the Matrix 003, but time spent above higher thresholds (>75 m*g*) were not. In contrast, Rowlands et al. (2019) found that the intensity gradient – a single metric describing the distribution of acceleration intensity across the 24-hour day (Rowlands et al., 2018) – was equivalent irrespective of accelerometer brand or wrist. The lack of equivalency in our study was most likely due to the 10% equivalence zone being too strict for less common activities that tend to have low magnitudes and/or high variability (Rowlands et al., 2019). This issue may have been exacerbated by the particularly low levels of higher-intensity physical activity typically observed in older adults.

Our findings, together with those of Rowlands et al. (2019), have implications for harmonising data across large-scale epidemiological cohorts that have used wrist-worn accelerometers. Historically, however, many studies have relied on waist-worn devices. In a systematic review of observational studies measuring device-based physical behaviours in adults, Pulsford et al. (2023) reported that the waist was the most common wear location, used in 53% of study waves compared to 20% for the wrist. Nonetheless, wear time compliance was higher for wrist-worn devices than for waist-worn ones, supporting the increasing preference for wrist placement in more recent studies. Furthermore, some studies used thigh-worn accelerometers (5% of study waves), which enable more accurate determination of posture and stepping, while others used multiple wear locations (13% of study waves) to measure different dimensions of physical behaviour, such as intensity, posture, and biological state (e.g., asleep or awake).

Substantial differences in accelerometer outputs have been observed between wear locations under free-living conditions. For example, a meta-analysis by Gall, Sun, and Smuck (2022) reported that wrist-worn accelerometers recorded, on average, 3,537 more steps per day than waist-worn ones. Wrist-worn devices also captured more time in MVPA and less sedentary time compared to waist-worn ones. Similarly, Maylor et al. (2023) found that the wrist-worn Axivity AX3 recorded higher average acceleration and a wider range of acceleration values than the thigh-worn activPAL micro.

Therefore, we are currently conducting two new free-living validation studies – in adults aged 18-30 years and those aged 40 years or older – to establish whether the same machine learning algorithms can be applied to different accelerometer wear locations, or if adjustment factors or location-specific algorithms are needed for data harmonisation.

To our knowledge, this is the first study to show that the same machine learning algorithms can be applied to different brands of accelerometer in different populations. However, the cross-nationality differences observed support the findings of two previous free-living validation studies in British and Chinese adults: CAPTURE-24 (Walmsley et al., 2021) and CAPTURE-24CN (Chen et al., 2023), respectively. In CAPTURE-24, 82% of all MVPA instances involved walking or cycling because almost all of the participants were office workers. In contrast, in CAPTURE-24CN, only 42% of MVPA instances involved walking or cycling, while the rest consisted of farm or construction work (Chen et al., 2023). Therefore, our finding that equivalency between the two accelerometer brands was lower for time spent in both LPA and MVPA but higher for overall daily step count in Chinese compared to British adults may be due to the UK-derived machine learning algorithms being better at identifying walking and cycling than farm or construction work.

Reprocessing the raw acceleration data from Chinese adults using a China-derived machine learning algorithm (Chen et al., 2023) improved equivalency between the two accelerometer brands for time spent in MVPA, bringing it closer to the level of equivalency observed in British adults (Supplementary Table S4). However, it still fell outside the predefined 10% equivalence zone, and equivalency for time spent in LPA was not improved by the China-derived algorithm. These findings highlight the need for further algorithm retraining using the additional wearable camera data collected in the current study.

It was somewhat surprising that equivalency between the two accelerometer brands for overall daily step count was higher in Chinese than in British adults, given that the *stepcount* machine learning algorithm was trained on data from British adults. Koffman and Muschelli (2024) applied five open-source and one proprietary algorithm to three publicly available datasets with ground truth step counts, including the free-living OxWalk dataset used to train the *stepcount* algorithm. They found that the *stepcount* algorithm was the most accurate, achieving the highest F1 score (0.89 ± 0.11) and the lowest mean absolute percentage error (8.6 ± 9.0%). However, the adults in OxWalk were much younger than those in the current study (mean age: 38.5 years vs. 65.6 and 66.7 years), highlighting the need for additional free-living validation studies across a wider range of ages and nationalities.

The sleep outcomes had the lowest ICCs in the current study, with the Matrix 003 recording more time in bed, higher overnight sleep duration, and poorer sleep efficiency than the Axivity AX3. This was particularly the case in Chinese adults, which may have been due to the shorter monitoring period of only two nights. Yuan et al. (2024) previously found that at least three days were required for stable weekly sleep parameter estimates. Further investigation using the additional sleep diary data collected is needed before pooling or comparing sleep outcomes from these two different accelerometer brands.

### Strengths and limitations

A strength of this study was the concurrent wear of two different brands of accelerometer (the well-established Axivity AX3 and the newly developed Matrix 003) in a random positional order on the dominant wrist 24 hours per day for up to eight days. Furthermore, the raw acceleration data from both accelerometer brands were processed identically using three well-validated, open-source machine learning algorithms to extract meaningful physical activity and sleep outcomes (Walmsley et al., 2021; Small et al., 2024; Yuan et al., 2024). Another strength of this study was that it was conducted in both the UK and China, allowing us to identify some important cross-nationality differences. The same methods were used in both countries, except the accelerometers were distributed and returned via post in the UK but in person in China. The monitoring period was also shorter in China than in the UK (two nights and one day versus eight days), meaning the findings may be less robust. Another limitation of this study was the use of self-selected cohorts, which may limit the generalisability of the findings. Moreover, older adults may exhibit substantially different physical activity and sleep patterns compared to middle-aged and younger adults.

### Conclusions

In both British and Chinese adults, average acceleration – a widely used measure of overall physical activity – was equivalent between the Axivity AX3 and the Matrix 003 worn in a random positional order on the dominant wrist. This finding suggests that data from these two different brands of accelerometer can be pooled and/or compared, including across large-scale epidemiological cohorts such as the UK Biobank, China Kadoorie Biobank, ELSA, and CHARLS. The vast majority of machine learning-derived physical activity and sleep outcomes were also equivalent or borderline equivalent. However, the cross-nationality differences observed likely stem from the UK-developed algorithms being better at identifying walking and cycling than farm or construction work. This highlights the need for further population-specific algorithm development and validation.

## Supplementary Material

Supplementary Materials

## Figures and Tables

**Figure 1:**
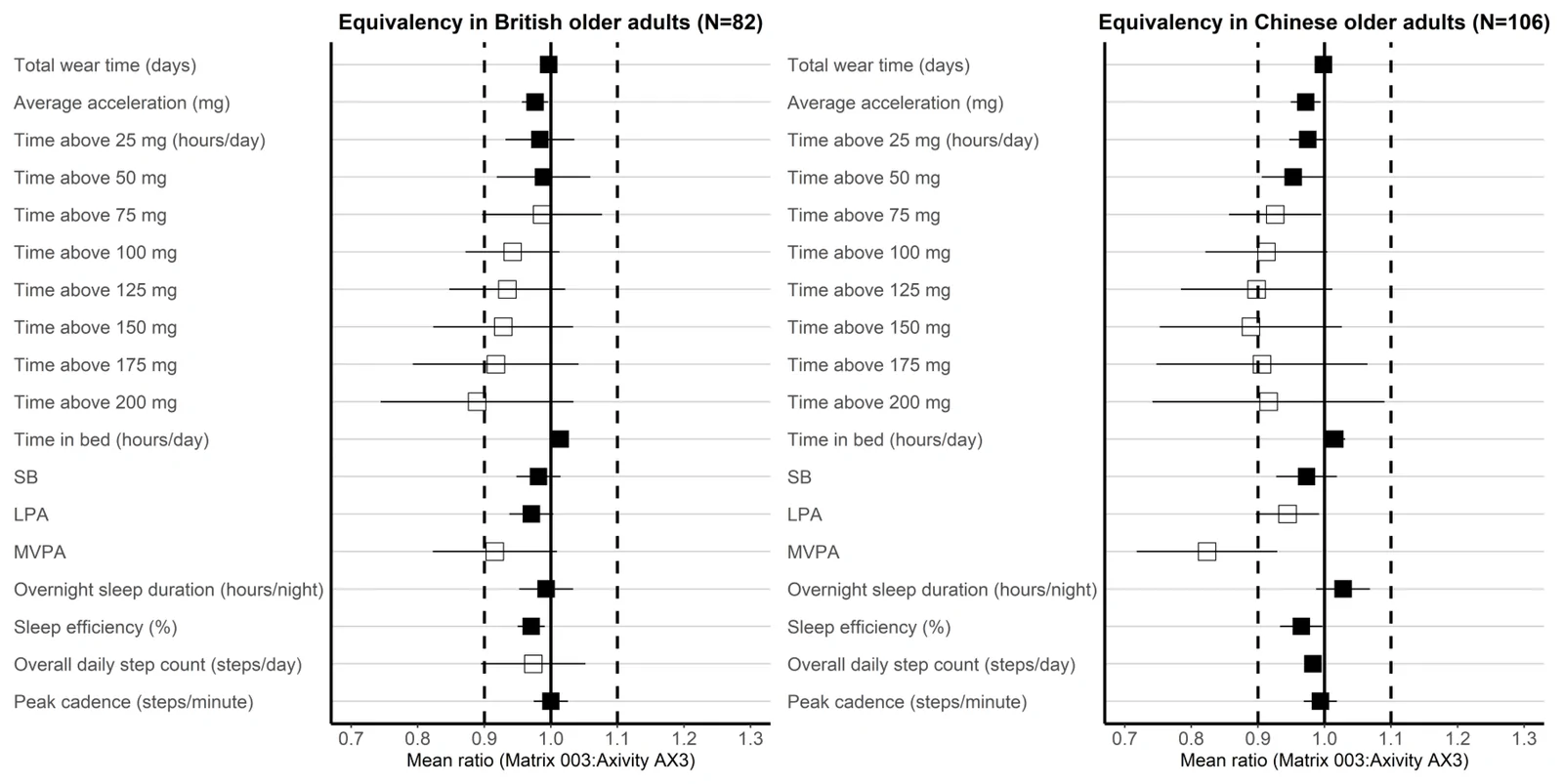
Equivalency between accelerometer brands for the physical activity and sleep outcomes by nationality. For equivalency, the dashed lines indicate the a priori 10% equivalence zone. The horizontal black lines show 95% CIs. Markers are denoted in solid squares if the 95% CI for the mean of the Matrix 003 accelerometer fell within ±10% of the mean of the Axivity AX3 accelerometer, otherwise markers are denoted in hollow squares. m*g* = milli-gravity units (dynamic acceleration expressed as ENMO). SB, sedentary behaviour; LPA, light physical activity; MVPA, moderate-vigorous physical activity; 95% CI, 95% confidence interval; ENMO, Euclidean Norm Minus One.

**Figure 2:**
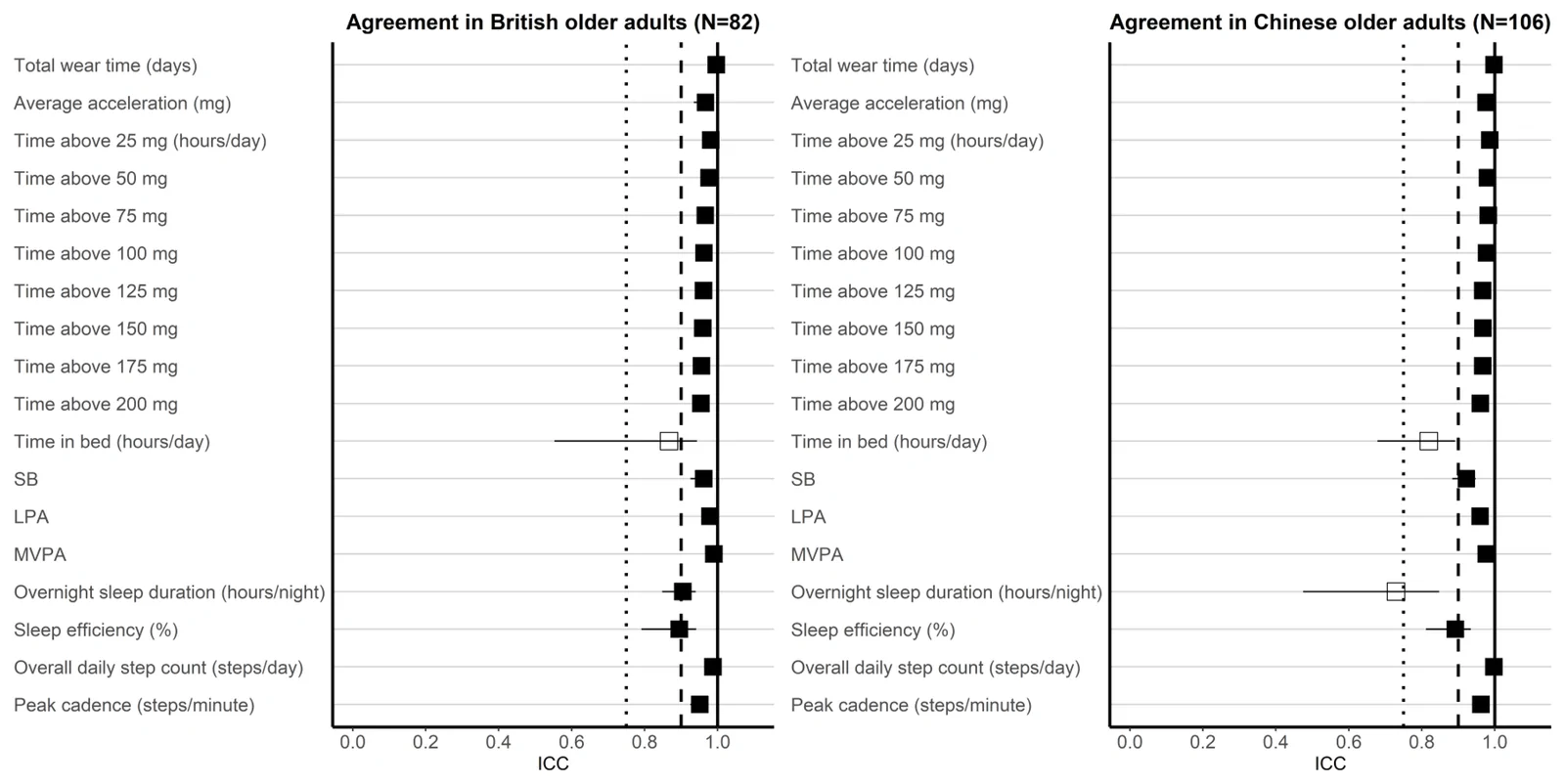
Agreement between accelerometer brands for the physical activity and sleep outcomes by nationality. For agreement, the dotted line represents ICCs of 0.75 (good reliability) and the dashed line represents ICCs of 0.9 (excellent reliability). The horizontal black lines show 95% CIs. Markers are denoted in solid squares if the lower band of the 95% CI of the ICC was >0.75, otherwise markers are denoted in hollow squares. m*g* = milli-gravity units (dynamic acceleration expressed as ENMO). SB, sedentary behaviour; LPA, light physical activity; MVPA, moderate-vigorous physical activity; ICC, intraclass correlation coefficient; 95% CI, 95% confidence interval; ENMO, Euclidean Norm Minus One.

**Table 1:** Physical activity and sleep outcomes by nationality and accelerometer brand.

	British adults (N=82)			Chinese adults (N=106)	
	Axivity AX3	Matrix 003	*P*-value	Axivity AX3	Matrix 003
Total wear time (days)	6.8 (6.6-7.0)	6.9 (6.6-7.0)	**0.041**	1.6 (1.5-1.6)	1.6 (1.5-1.6)
Average acceleration (m*g*)	27.9 (23.5-34.5)	26.8 (21.7-32.4)	**<0.001**	25.8 (19.7-34.6)	26.1 (19.2-32.7)
Time above 25 m*g* (hours/day)	6.9 (1.7)	6.8 (1.7)	**0.001**	7.5 (6.0-9.2)	7.7 (5.8-8.9)
Time above 50 m*g*	4.3 (1.4)	4.2 (1.4)	0.162	4.1 (3.0-5.5)	4.4 (2.8-5.7)
Time above 75 m*g*	2.6 (1.1)	2.6 (1.1)	0.277	2.3 (1.2-3.4)	2.3 (1.3-3.4)
Time above 100 m*g*	1.5 (1.1-2.2)	1.6 (1.0-2.3)	0.594	1.2 (0.6-2.0)	1.3 (0.6-2.0)
Time above 125 m*g*	1.0 (0.6-1.5)	1.0 (0.6-1.4)	0.776	0.7 (0.3-1.2)	0.6 (0.3-1.3)
Time above 150 m*g*	0.6 (0.4-1.0)	0.6 (0.3-1.0)	0.876	0.3 (0.1-0.8)	0.4 (0.2-0.8)
Time above 175 m*g*	0.4 (0.2-0.7)	0.4 (0.2-0.7)	0.746	0.2 (0.1-0.5)	0.2 (0.1-0.5)
Time above 200 m*g*	0.3 (0.1-0.5)	0.2 (0.1-0.5)	0.495	0.1 (0.0-0.4)	0.2 (0.0-0.4)
Time in bed (hours/day)	7.7 (7.1-8.3)	7.8 (7.3-8.5)	**<0.001**	7.0 (6.3-8.2)	7.3 (6.6-8.7)
SB	10.8 (1.8)	10.6 (1.7)	**<0.001**	10.6 (2.8)	10.3 (2.5)
LPA	4.4 (3.5-5.0)	4.3 (3.4-5.1)	**0.001**	4.5 (3.4-6.2)	4.6 (3.2-6.2)
MVPA	1.1 (0.6-1.6)	1.0 (0.6-1.6)	0.077	1.0 (0.5-1.9)	1.0 (0.4-1.9)
Sleep duration (hours/night)	5.3 (3.8-6.3)	6.0 (3.9-6.9)	**0.001**	5.5 (4.3-6.3)	6.1 (5.3-6.8)
Sleep efficiency (%)	75.1 (58.5-83.8)	70.7 (48.4-79.9)	**<0.001**	85.9 (78.1-92.3)	83.3 (73.9-88.3)
Step count (steps/day)	11,018 (4,138)	10,726 (3,995)	**<0.001**	10,409 (7,686-15,903)	10,534 (7,005-15,343)
Peak cadence (steps/minute)	116 (13)	116 (14)	1.000	107 (14)	106 (14)

Mean (SD) is presented where data were normally distributed, otherwise median (25^th^-75^th^ percentile). m*g* = milli-gravity units (dynamic acceleration expressed as ENMO). SB, sedentary behaviour; LPA, light physical activity; MVPA, moderate-vigorous physical activity; SD, standard deviation; ENMO, Euclidean Norm Minus One.

## Data Availability

The dataset analysed during the current manuscript is not publicly available due to institutional data sharing restrictions, but we anticipate anonymised data to be shared in future as part of a wider project.

## Highlights

1) The harmonisation of accelerometry data across large-scale epidemiological cohorts is currently being hindered by different brands and processing methods.
2) Average acceleration – a widely used measure of overall physical activity – was comparable between the Axivity AX3 and the Matrix 003 in both British and Chinese adults aged 50 years or older; this suggests that data from these two different brands of wrist-worn accelerometer can be pooled and/or compared.
3) Machine learning-derived physical activity and sleep outcomes were also largely comparable; however, the cross-nationality differences observed highlight the need for further population-specific algorithm development and validation.
